# Altering PTPRD via genetics or pharmacology modulates 3xTg-AD mouse neurofibrillary pathology

**DOI:** 10.3389/fnins.2026.1803332

**Published:** 2026-03-31

**Authors:** George R. Uhl, Balaji Kannan, Sarah Jung, Joungil Choi, Ian Henderson, Kevin Schultz

**Affiliations:** 1 Neurology Service, VA Maryland Healthcare System, Baltimore, MD, United States; 2 Department of Neurology, University of Maryland School of Medicine, Baltimore, MD, United States; 3 Research Service, VA Maryland Healthcare System, VAMHCS, Baltimore, MD, United States; 4 Department of Internal Medicine, University of New Mexico School of Medicine, Albuquerque, NM, United States; 5 Department of Chemistry, Goucher College, Baltimore, MD, United States

**Keywords:** flavonols, mouse model, neurofibrillary tangles, novel therapeutics, senile plaques, substrate selective positive allosteric modulator

## Abstract

Densities of neurofibrillary tangles (NFTs), a major Alzheimer’s disease (AD) pathology, display genetic associations with variants in the receptor type protein tyrosine phosphatase D (PTPRD) gene. NFTs are rich in tau protein that is hyperphosphorylated, prominently by the glycogen synthase kinases (GSK) 3α/β. PTPRD dephosphorylates GSK3s, reducing their activities and providing an attractive candidate molecular mechanism for PTPRD/NFT associations. We have used AT-8 and Aβ immunohistochemistry to assess hyperphosphorylated tau/NFT and Aβ/senile plaque pathologies, developed and characterized 3xTg-AD mice with wildtype or reduced PTPRD expression and assessed results of treatments with our (a) PTPRD phosphatase inhibitor pentilludin, (b) lead PTPRD positive allosteric modulator (PAM) quercetin and (c) drug candidate PTPRD PAM active metabolite 6BrQ. Four-month 3xTg-AD/PTPRD +/− mice display AT-8 immunoreactivity in hippocampal neurons, much earlier than 3xTg-AD/PTPRD +/+ mice. There are modest effects of reducing PTPRD expression on densities of Aβ/senile plaque structures assessed at 21 months. 3xTg-AD (but not wildtype C57) mice treated (weeks 6–16) with pentilludin display abundant hyperphosphorylated tau at 4 months. 3xTg-AD/PTPRD+/− mice treated (weeks 6–16) with quercetin or 6BrQ display >50% and >95% reductions in AT-8 immunoreactive hippocampal neuron counts, respectively. These results support roles for PTPRD in AD neurofibrillary pathophysiology and for orally-bioavailable drugs that can be metabolized to 6BrQ to slow development of this pathology.

## Background

Brains of individuals dying with Alzheimer’s disease (AD) display neurofibrillary tangles (NFTs) rich in hyperphosphorylated tau protein and senile plaques rich in the Aβ fragment cleaved from the amyloid precursor protein (DeTure and Dickson, 2019; Sery et al., 2013). Densities of NFTs in AD brains display molecular genetic associations with variants in the gene encoding PTPRD (the receptor type protein tyrosine phosphatase D) (Chibnik et al., 2018; Drgonova et al., 2015). There is specificity: densities of senile plaques are not associated with PTPRD genomic variants (Chibnik et al., 2018).

A mechanistic hypothesis for these associations posits that decreased PTPRD expression reduces PTPRD’s ability to downregulate principal tau hyperphosphorylating enzymes, glycogen synthase kinase 3 (GSK3) α and β (Cavallini et al., 2013), via dephosphorylation of their activity-regulating (Cavallini et al., 2013; Hughes et al., 1993) phosphotyrosines 276 and 216, respectively (Krishnankutty et al., 2017). This hypothesis has received indirect support in humans: (a) the prominent dietary flavonol quercetin provides positive allosteric modulation (PAM) of PTPRD’s ability to dephosphorylate glycogen synthase kinase 3 (GSK3) α/β (Henderson et al., 2022a) and (b) higher dietary intake of quercetin and other flavonols is associated with decreased AD incidence, especially in individuals at higher genetic risk for AD (Holland et al., 2020; Jennings et al., 2024; Shishtar et al., 2020; Uhl et al., 2025a).

A widely-used AD model, the 3xTg-AD mouse, contains pathogenic variants in tau [initially identified in a family with frontotemporal dementia (Mirra et al., 1999)], the amyloid precursor protein (APP) and the APP-processing enzyme presenilin (Oddo et al., 2003; Belfiore et al., 2019). These mice develop pathologically-hyperphosphorylated tau and Aβ/senile plaque-like deposits over a well-characterized 21-month time-course (Belfiore et al., 2019). Quercetin reduces development of AD-like pathology in these 3xTg-AD mice if it is administered during the 3 months (when administered intraperitoneally) or 12 months (when administered orally) prior to their sacrifice at ages 18–21 months (Paula et al., 2019; Sabogal-Guaqueta et al., 2015).

These human, mouse model and *in vitro* data suggest novel approaches to: (a) improved modeling of the development of AD neurofibrillary pathology, (b) validating human NFT associations with PTPRD genomic variation and (c) testing compounds to reduce the accumulation of tau pathology. In particular, 3xTg-AD mice with reduced PTPRD expression could provide a testbed to elucidate effects of quercetin and an analog, 6-bromoquercetin (6BrQ), that we have identified during structure–activity relationship studies of PTPRD PAM activities of quercetin analogs. Here, we thus test the hypotheses that (a) genetic or pharmacologic reduction in PTPRD activity will hasten and enhance the accumulation of neurofibrillary tau pathology in mouse models, (b) lead compound and improved PTPRD positive allosteric modulators will reduce the *in vivo* accumulation of neurofibrillary pathology. We discuss our results in the context of the behavioral phenotypes and downstream signaling mechanisms related to manipulating PTPRD as well as the other targets and off targets that could be engaged by quercetin and 6BrQ.

## Methods

3xTg-AD mice [JAX (004807 B6); 129-Tg (APPSwe, tauP301L)1Lfa Psen1tm1Mpm/Mmjax] were crossed with heterozygous PTPRD knockout mice housed in the VA Maryland Healthcare System AALAC-certified animal facility under IACUC-approved protocols. Pups were genotyped as described (Drgonova et al., 2015; Belfiore et al., 2019). C57/bl mice were purchased (Charles River).

**Figure 1:**
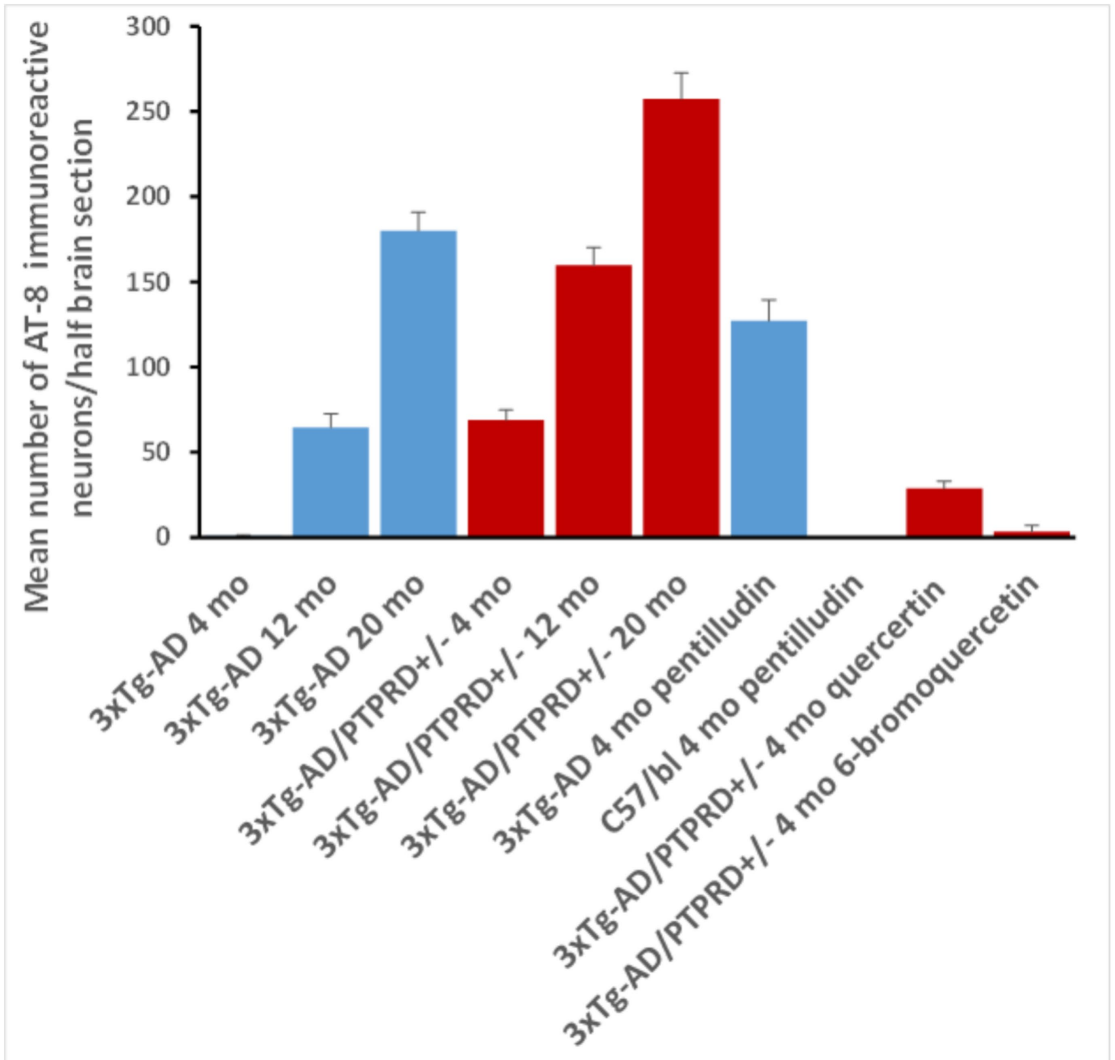
Numbers of hippocampal neurons displaying AT8 immunoreactive hyperphosphorylated tau in mice with genotypes, treatments and ages at sacrifice shown. Blue bars: 3xTg-AD with wildtype PTPRD. Red bars: 3xTg-AD/PTPRD +/−. Numbers are averages in half brain sections taken from −3.08 and −3.8 mm from bregma, +/− SEM with *n* = 6, 4, 4, 8, 4, 6, 6, 6, 8 and 6, respectively. Overall ANOVA *p* = 3.1 × 10^−47^ (df 9). All Tukey–Kramer *post-hoc* comparisons are significant *p* < 0.05.

Some mice were randomly assigned to treatments with (a) PEG600 vehicle, (b) pentilludin (NetChem, >92% pure); 100 mg/kg po/gavage every other day (qod) from week 6 of age until sacrifice at 16 weeks, (c) quercetin (Sigma), 25 mg/kg ip qod or (d) 6-bromo quercetin (6BrQ) (>97% pure; synthesized and purified as described below), 25 mg/kg ip qod. Sample sizes (*n* = 4–8, Figure 1) were based on breeding success and effect sizes identified in studies of quercetin effects in older 3xTg-AD mice (Paula et al., 2019; Sabogal-Guaqueta et al., 2015).

Mice of the appropriate genotypes, ages and treatments were sacrificed by perfusion with buffered 4% depolymerized paraformaldehyde under anesthesia. Seven micromolar coronal sections from paraffin-embedded brains (levels −3.08 and −3.8 mm from bregma) were stained by heating to 58 °C for 1 h, cooling, deparaffining in xylene, rehydrating with graded alcohols, incubations in 10 mM sodium citrate, pH 6.0, 3% H_2_O_2_, mouse IgG blocker and AT8 antibody [mouse monoclonal, Thermo Fisher MN1020, 1.25 μg/mL that can recognize hyperphosphorylated human (Goedert et al., 1995) or mouse (Clodfelder-Miller et al., 2006) tau protein] or anti-Aβ antibody (mouse monoclonal, Sigma MABN10, 1:2,000 dilution) overnight at 4 °C followed by washing. Sections were incubated with M.O.M. biotinylated anti-mouse IgG (1:250), avidin-biotin complex/horseradish peroxidase (Vector) and developed with diaminobenzidine.

Numbers of phosphotau/AT8-immunoreactive hippocampal neurons and of Aβ-immunoreactive elements in hippocampus and overlying cortex were quantitated in half brain stitches obtained with automated slide scanning via a Zeiss AxioImager M2 with a 10× 0.3 NA objective with analysis using ImageJ by an investigator blinded to genotype and treatment. Results presented average neuronal counts at −3.08 and −3.8 mm from bregma. Analyses of variance were followed by Tukey–Kramer *post-hoc* tests using Graph Pad Prism software. Data is available from the authors.

6 bromo quercetin (6BrQ) was synthesized n-Bromosuccinimide (0.605 g, 3.4 mmol) was dissolved in deionized water (200 mL) containing 6 mL of 3 M NaOH. This solution was added dropwise to quercetin (1.0 g, 3.31 mmol) in methanol (200 mL) under nitrogen. After 8 h of stirring at room temperature, sodium hydrosulfite (2.0 g, 11.5 mmol in 100 mL deionized water) was added. The reaction mixture was acidified using 3 M HCl and cooled overnight. The resulting yellow precipitate was collected by filtration and dried to give a crude product (0.807 g, 68% yield). Four hundred milligrams of the crude product was purified by flash silica column chromatography using a gradient of 70 to 90% diethyl ether/hexanes [*R*_f_ = 0.40 (90% diethyl ether/hexanes)] to afford a yellow powder (0.168 g, 42% yield). Three hundred megahertz NMR verification of structure and purity came from 1H NMR in (400 MHz) d6-acetone *δ* = 6.75 (s, 1H), 7.00 (d, *J* = 8.4 Hz, 1H), 7.70 (dd, *J* = 8.4, 2.1 Hz, 1H), 7.83 (d, *J* = 2.1 Hz, 1H), 13.05 (s, 1H), 1 H NMR in (400 MHz) DMSO *δ* = 6.62 (s, 1H), 6.89 (d, *J* = 8.4 Hz, 1H), 7.56 (dd, *J* = 8.4, 2.1 Hz, 1H), 7.68 (d, *J* = 2.1 Hz, 1H), 9.37 (s, 1H), 9.59 (s, 1H), 9.67 (s, 1H), 11.70 (s, 1H), 13.40 (s, 1H) and 13 C NMR in (100 MHz) d-acetone δ = 92.94, 94.77, 104.42, 115.80, 116.21, 121.49, 123.52, 136.73, 145.67, 147.60, 148.52, 156.03, 158.78, 160.91, 176.05.

## Results

### Genetic or pharmacologic reduction in PTPRD activity hastens the accumulation of neuronal neurofibrillary pathology in mouse models

#### 3xTg-AD mice

**Figure 2:**
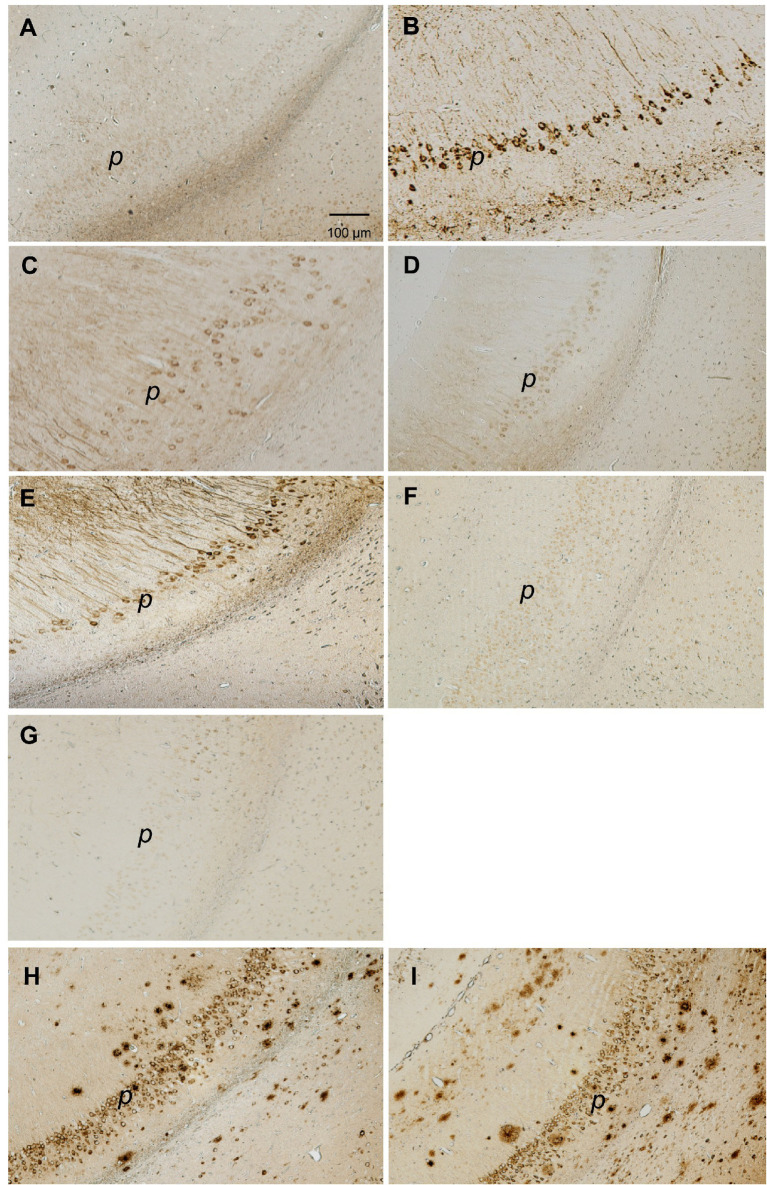
Photomicrographs of hippocampal AT8 phosphotau **(A–G)** and Aβ plaque-like **(H–I)** immunoreactivities (−3.8 mm from bregma). **(A)** 4-month-old 3xTg-AD. **(B)** 20-month-old 3xTg-AD. **(C)** 4-month-old 3xTg-AD/PTPRD+/−. **(D)** 4-month-old 3xTg_AD/PTPRD +/− treated with quercetin (25 mg/kg qod ip weeks 6–16). **(E)** 4-month-old 3xTg-AD mouse treated with pentilludin (100 mg/kg qod po weeks 6–16). **(F)** 4-month old C57bl (wildtype) mouse treated with pentilludin (100 mg/kg qod po weeks 6–16). **(G)** 4-month old 3xTg-AD/PTPRD+/− mouse treated with 6BrQ (25 mg/kg qod weeks 6–16). **(H)** 20-month old 3xTg-AD. **(I)** 20-month old 3xTg-AD/PTPRD +/−. Bar 100 μM (for all photomicrographs shown).

20 month-old 3xTg-AD mice with wildtype PTPRD display pathologically-hyperphosphorylated tau. This is manifest as immunoreactivity that fills much of the perikarya of hippocampal neurons using the AT-8 monoclonal antibody that selectively recognizes this pathologically-hyperphosphorylated tau (Goedert et al., 1995; Malia et al., 2016). We observe this immunoreactivity in sections taken at −3.08 and at −3.8 mm from bregma, as previously reported (Belfiore et al., 2019). There are 3 AT8-immunoreactive hippocampal neurons/half brain section on average in 3xTg-AD mice sacrificed at 4 months, 65 at 12 months and 180 at 20 months, consistent with prior reports (Belfiore et al., 2019) (Figures 1, 2). Each of these comparisons achieves significance in Tukey–Kramer *post-hoc* testing.

#### 3xTg-AD/PTPRD +/− mice

3xTg-AD mice with reduced PTPRD expression (3xTg-AD/PTPRD +/− mice) are fertile. Many (more females) survive to 20 months of age. These mice display 69 AT8-immunoreactive hippocampal neurons/half brain section at 4 months, 160 at 12 months and 258 at 20 months, numbers that are each greater than those in 3xTg-AD mice with wildtype PTPRD sacrificed at these ages (Figures 1, 2). Each of these comparisons achieves significance in Tukey–Kramer *post-hoc* testing.

#### Pentilludin-treated 3xTg-AD mice

There are effects of pharmacologic reductions in PTPRD activity using pentilludin (Henderson et al., 2022b; Uhl, 2023) [100 mg/kg po (gavage), alternating days (qod) weeks 6–16]. Pentilludin-treated 3xTg-AD mice display 127 AT8-immunoreactive hippocampal neurons/half brain section at 4 months of age, while vehicle-treated mice display 6 (ANOVA *p* = 2 × 10^−10^). There are no AT8 immunoreactive neurons in 4 month-old pentilludin-treated wildtype C57bl mice (*p* = 2 × 10^−10^ vs. pentilludin effects in 3xTg-AD mice) (Figures 1, 2). Cage behaviors of these mice appeared normal. Neither observations of these animals nor observations during pentilludin dosing to mice or rats support significant general toxicity from this dose level, including formal “Irwin” behavioral battery in acutely and repeatedly-dosed rats (Henderson et al., 2022b Uhl et al., 2025b).

### Genetic or pharmacologic reduction in PTPRD activity has more modest effects on accumulation of Aβ-immunoreactive senile-plaque like structures

**Figure 3:**
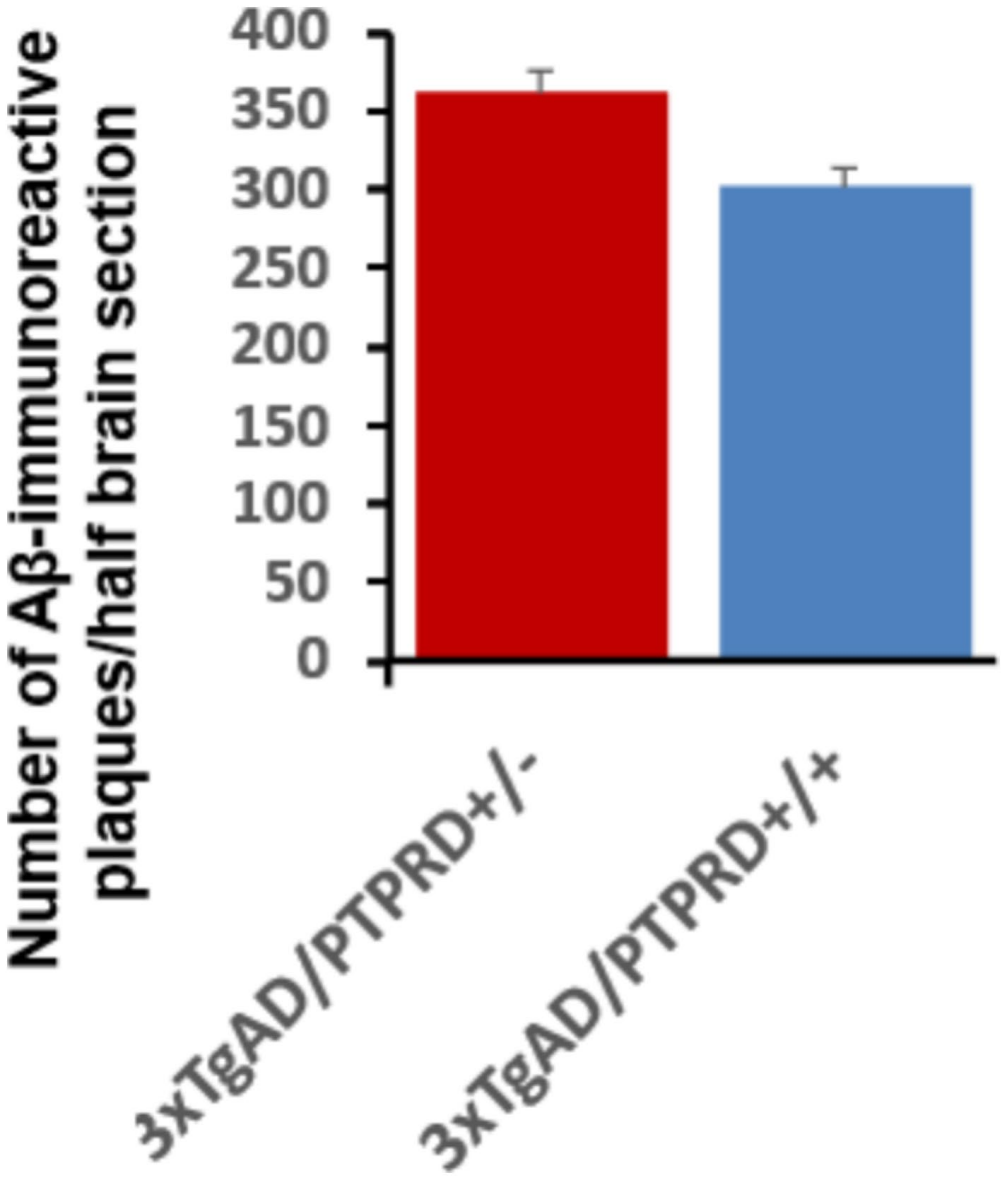
Effects of genetically-reduced PTPRD expression on development of Aβ immunoreactive plaque-like structures. Mean numbers (+/− SEM) of hippocampal and cortical Aβ immunoreactive elements in half brain sections from −3.08 and −3.8 mm from bregma in 20-month-old mice with genotypes indicated.

There are much more modest effects of altered PTPRD expression on deposition of the prominent amyloid constituent, Aβ, observed using a monoclonal antibody that selectively recognizes Aβ (Figures 2H,I, 3). No Aβ-immunoreactive elements are noted in hippocampus or overlying cortex of 4 month-old 3xTg-AD mice with any PTPRD genotype or in pentilludin-treated 3xTg-AD mice. Few are seen at 12 months. At 20 months, 3xTg-AD mice with wildtype PTPRD display 301 Aβ-immunoreactive plaque-like structures/half brain section of hippocampus and overlying cortex while 3xTg-AD/PTPRD +/− mice display 361, a difference that achieves statistical significance (Figures 2H,I, 3; ANOVA *p* = 5 × 10^−4^).

### Quercetin reduces AT8 immunoreactive neuronal phosphorylated tau accumulation in 3xTg-AD/PTPRD +/− mice

**Figure 4:**
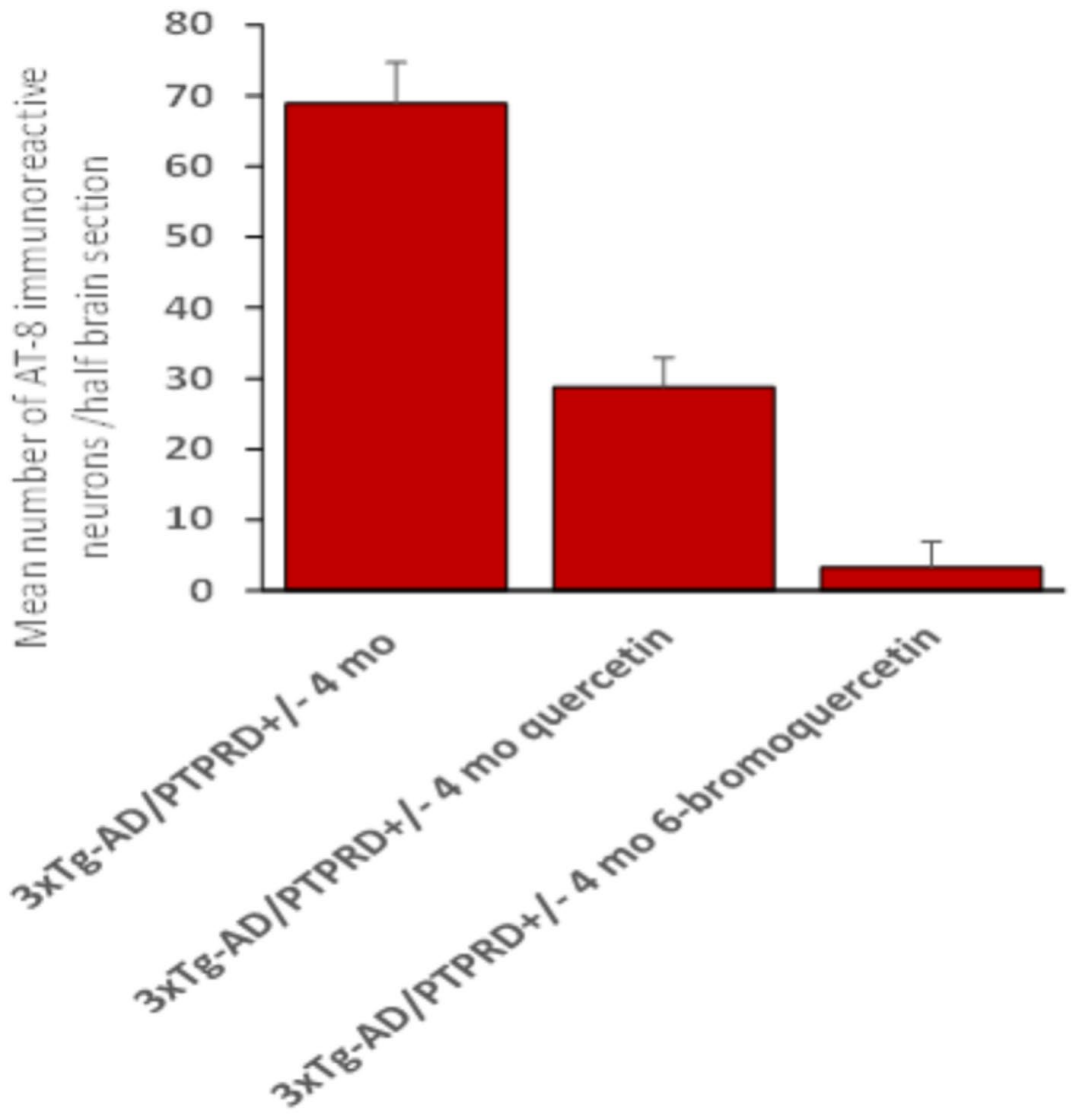
Effects of treatment with PTPRD PAMs. Mean numbers (+/− SEM) of hippocampal AT-8-immunoreactive neurons/half brain section from −3.08 and −3.8 mm from bregma in 4-month-old 3xTg-AD/PTPRD +/− mice treated with vehicle, quercetin or 6-BrQ (25 mg/kg ip, qod) from weeks 6–16 of age.

To test effects of our lead compound PTPRD PAM, quercetin, in our new model, we administered quercetin 25 mg/kg/alternate day, ip, to 3xTg-AD/PTPRD +/− mice from weaning to 4 months of age (weeks 6–16). There were no stigmata of any overall toxicity in observations of these mice and their cage behaviors. Quercetin treatment reduced the accumulation of hyperphosphorylated tau in hippocampal neurons in these mice. There were 29 AT8 immunoreactive hippocampal neurons in quercetin-treated 4 month-old 3xTg-AD/PTPRD +/− mice vs. the 69 AT8-positive neurons found in vehicle-treated 3xTg-AD/PTPRD+/− mice (Figures 2, 4; ANOVA *p* = 8 × 10^−9^).

### The improved PTPRD PAM 6-bromo quercetin (6BrQ) reduces AT8 immunoreactive neuronal phosphorylated tau accumulation in 3xTg-AD/PTPRD +/− mice

We also treated 3xTg-AD/PTPRD +/− mice with 25 mg/kg/alternate day of 6BrQ ip from weeks 6–16. There was no sign of overall toxicity in the 6Br-Q-treated mice. There were 3 AT8 immunoreactive neurons/half brain section in these mice. These improved results were significantly different from those obtained in either vehicle or quercetin-treated 3xTg-AD/PTPRD +/− mice (Figures 2, 4; ANOVA *p* = 5.6 × 10^−8^ overall with Tukey–Kramer *q* = 21 and 8.1, respectively vs. critical value 4.69).

## Discussion

Combined mouse and human genetic and pharmacologic results, taken together, support sizable roles for PTPRD in AD neurofibrillary pathophysiology. Our present and prior results encourage the idea that glycosylated 6BrQ analogs could reduce human AD pathophysiology.

Our finding that adding reduced PTPRD expression hastens development of neurofibrillary pathology in 3xTg-AD mice fits well with human genetic associations (Chibnik et al., 2018; Drgonova et al., 2015). Lifelong differences in levels of PTPRD expression thus alter development of neurofibrillary pathology in 3xTg-AD mice and, likely, in humans.

Results of pharmacological studies with our potent, irreversible PTPRD inhibitor pentilludin (Henderson et al., 2022b; Uhl, 2023) support and extend genetic results. We do not need lifelong reductions in PTPRD activity to produce substantial amounts of Alzheimer’s-like tau pathophysiology in mice with the 3xTg-AD background. Lack of any such pathology in pentilludin-treated wildtype C57bl mice points to the safety of pentilludin in animals without these disease variants. There is thus mutual support from parallel changes in tau pathophysiology when we reduce PTPRD activity by genetic or pharmacologic strategies in 3xTg-AD mice.

The data also provide evidence for specificity. Although there is a significantly greater accumulation of Aβ-immunoreactive senile-plaque-like pathology in 20-month-old 3xTg-AD/PTPRD +/− mice than in 3xTg-AD mice with wildtype PTPRD, 3xTg-AD mice with reduced PTPRD expression develop Aβ-immunoreactive senile-plaque-like pathology at about the same time as 3xTg-AD mice with wildtype PTPRD. These results are thus consistent with the lack of a strong association between human PTPRD variation and senile plaque densities in AD brains (Chibnik et al., 2018).

Our findings that week 6–16 quercetin administration substantially reduces development of neurofibrillary pathology in 3xTg-AD/PTPRD +/− mice add to prior observations concerning AD-related benefits of quercetin in 3xTg-AD mice (Paula et al., 2019; Sabogal-Guaqueta et al., 2015). These data help to validate our new mouse model. These quercetin results also accord well with the findings that higher human dietary flavonol intake reduces risk of developing AD or Alzheimer’s disease related dementias, especially in individuals who display higher genetic risk for AD (Holland et al., 2020; Jennings et al., 2024; Shishtar et al., 2020; Uhl et al., 2025a). Quercetin also has other actions of possible relevance for AD, including antinflammatory actions, inhibition of acetyl-cholinesterase and Aβ aggregation and, most discussed, antioxidant properties (Khan et al., 2019; Calderaro et al., 2022; Minocha et al., 2022). Quercetin (10^−7^ M) fails to exert any significant activity in a large panel of EUROFINS screens (Uhl et al., 2026). Our current results that suggest that PTPRD activities are likely to contribute prominently to flavonol activities in preventing AD-related pathologies and AD/ADRD.

Quercetin is not an optimal PTPRD PAM, however. We have sought improvements in quercetin PAM activity in other work, synthesizing and testing PTPRD PAM activities of 108 synthesized and purchased quercetin analogs. 6BrQ has PTPRD PAM activities, enhancing PTPRD’s release of orthophosphate from our pYGSK3 phosphopeptide better than quercetin while displaying nearly-identical antioxidant properties and no significant activity at 10^−7^ M in a large panel of EUROFINS screens (Uhl et al., 2026). More than 95% elimination of neurofibrillary neuronal pathology in mice treated from weaning to adulthood with 6BrQ, while quercetin provides only a 55% reduction, supports the *in vitro* data that identified 6BrQ as a better PTPRD PAM. These results also support development of a compound that provides 6BrQ, *in vivo*, to reduce development of AD neurofibrillary pathology.

Quercetin is also not an optimal drug for oral administration. It displays modest human bioavailability (Zhu et al., 2024; Almeida et al., 2018; Dabeek and Marra, 2019) and large individual differences in plasma levels attained following administration of the same doses to different individuals. By contrast, several glycosylated quercetin analogs, including those with 3 and 4′ glycosylation (Olthof et al., 2000), are much more orally bioavailable and appear to provide less individual variation in plasma levels observed following the same dose. Glycosylated quercetin is readily deglycosylated by gut endothelial cell luminal and/or cytoplasmic enzymes in ways that facilitate systemic delivery of deglycosylated quercetin (Dabeek and Marra, 2019). “6BrQG,” glycosylated at 3, 4′ or perhaps other positions, will thus likely provide a superior drug for oral administration.

Heterozygous PTPRD knockout mice display normal performance in cognitive testing while homozygotes display substantial deficits (Drgonova et al., 2015). We have noted trends toward reduced Morris water maze performance in aging 3xTg-AD/PTPRD +/− micethat have paralleled several of the neuropathological changes reported here but do not reach statistical significance, though differences from results in 3xTg-AD mice with homozygous PTPRD knockout do achieve statistical significance (Supplementary material) The greater variability in behavioral data has led us to focus on neuropathology developed by 4 months in this initial brief report.

We have identified candidate brain substrates for PTPRD, including glycogen synthase kinases (GSK3 β and α) based on brain phosphoproteomic results, coexpression with PTPRD and observation of orthophosphate release from the corresponding phosphopeptide by recombinant PTPRD phosphatase (Henderson et al., 2022a). While PTPRD effects on GSK3 activities provide likely contributors for the effects on tau phosphorylation identified in the present work, it is also possible that other PTPRD substrates might be involved as well. Our present results do fit with behavioral and other data that indicates that pentilludin engages PTPRD in brain (Uhl et al., 2025b).

The amyloid cascade hypothesis of AD supports a primary role for amyloid pathology in triggering secondary neurofibrillary/tau pathology (Hardy and Higgins, 1992). This hypothesis has helped to motivate production and testing of antibodies that recognize distinct forms of anti-Aβ pathology, aiming to reduce amyloid pathology in AD brains. Two of these antibodies are currently licensed for human use based on their activities in slowing rates of clinical progression of AD (van Dyck et al., 2023; Sims et al., 2023). Our results that document appearance of neuronal AT8 immunoreactivity well before we find Aβ in 3xTg-AD/PTPRD +/− mice do not fit with the temporal relationships for AD pathogenesis postulated by the initial strong “Aβ first” form of the amyloid cascade hypothesis. However, it seems likely that therapeutic strategies based on reduced tau phosphorylation could provide benefits that add to those of anti-Aβ antibodies in reducing AD pathophysiology and dementia.

Data from dietary studies suggest that benefits of flavonols and improved analogs, including, glycosylated 6BrQ, may well be greatest for those with higher genetic risks for AD and those whose baseline flavonol intake is low (Uhl et al., 2025a). Future studies will help to define individual characteristics, including genotypes, flavonol intake and brain/plasma biomarkers for phosphotau and Aβ, that will result in individuals’ optimal assignment to glycosylated 6BrQ drugs and antiamyloid therapies.

## Data Availability

The raw data supporting the conclusions of this article will be made available by the authors, without undue reservation.
