# Supplementary material for: Altering PTPRD via genetics or pharmacology modulates 3xTg-AD mouse neurofibrillary pathology
Source: Front Neurosci. 2026 Mar 31;20:1803332. doi: 10.3389/fnins.2026.1803332 (PMC13076478; doi:10.3389/fnins.2026.1803332)

Supplement

**Morris Water Maze:**

Water maze test was performed with 4 and 12 months old 3xTgAD/PTPRDs. A circular water pool of 2m in diameter was filled with water (23 ± 2 °C temperature). Different shapes were marked on the walls of the four quadrants, East (E), West (W), North (N) and South (S) of the pool and walls of the room for visual cues. A platform (10cm in diameter) was placed at the centre of the Northwestern (NW) quadrant and the platform location was not changed throughout the experiment. For 5 days learning period, water level was filled up to 1 cm below the platform surface and all the mice were trained to find the platform 4 trails a day for 6 consecutive days. Mice that gently dropped into the pool found the platform by observing the spatial cues within 95 sec were left on the platform for 10 s for learning. Mice that did not find in 95sec were placed on the platform for 10 s. On day 7, mice were tested in water maze after removing the platform. For mice visibility testing on day 8, non-toxic, odourless white tempera paint was added to make the water opaque and mice were introduced in to the pool without platform. For all the 8 days mice swimming path, time spent in each quadrant, number of attempts to find the platform or crossings in the platform zone and time spent in search of the platform in the NW quadrant were recorded using ANY-maze software(Stoelting, USA).

**Supplement Figure 1 legend:**

Age-dependent spatial learning and behavioural test by Morris Water Maze comparing 4 and 12 month old 3xTg-AD/PTPRD+/+; +/- and -/- mice. (**A**) Representative images of the trajectory in the MWM on Day 7; (**B**) Time spent in all the 4 quadrants; (**C**) Latency to find the platform in the 6 days of training, (**D**) Number of crossings in the attempt to find the platform and (**E**) Day 8 visibility test on opaque water showing number of investigation to the platform zone. Data are expressed as mean ± SEM, with sample sizes ranging from 4 to 26. Significance levels are indicated as follows: *p < 0.05; **p < 0.005; ***p < 0.0005.


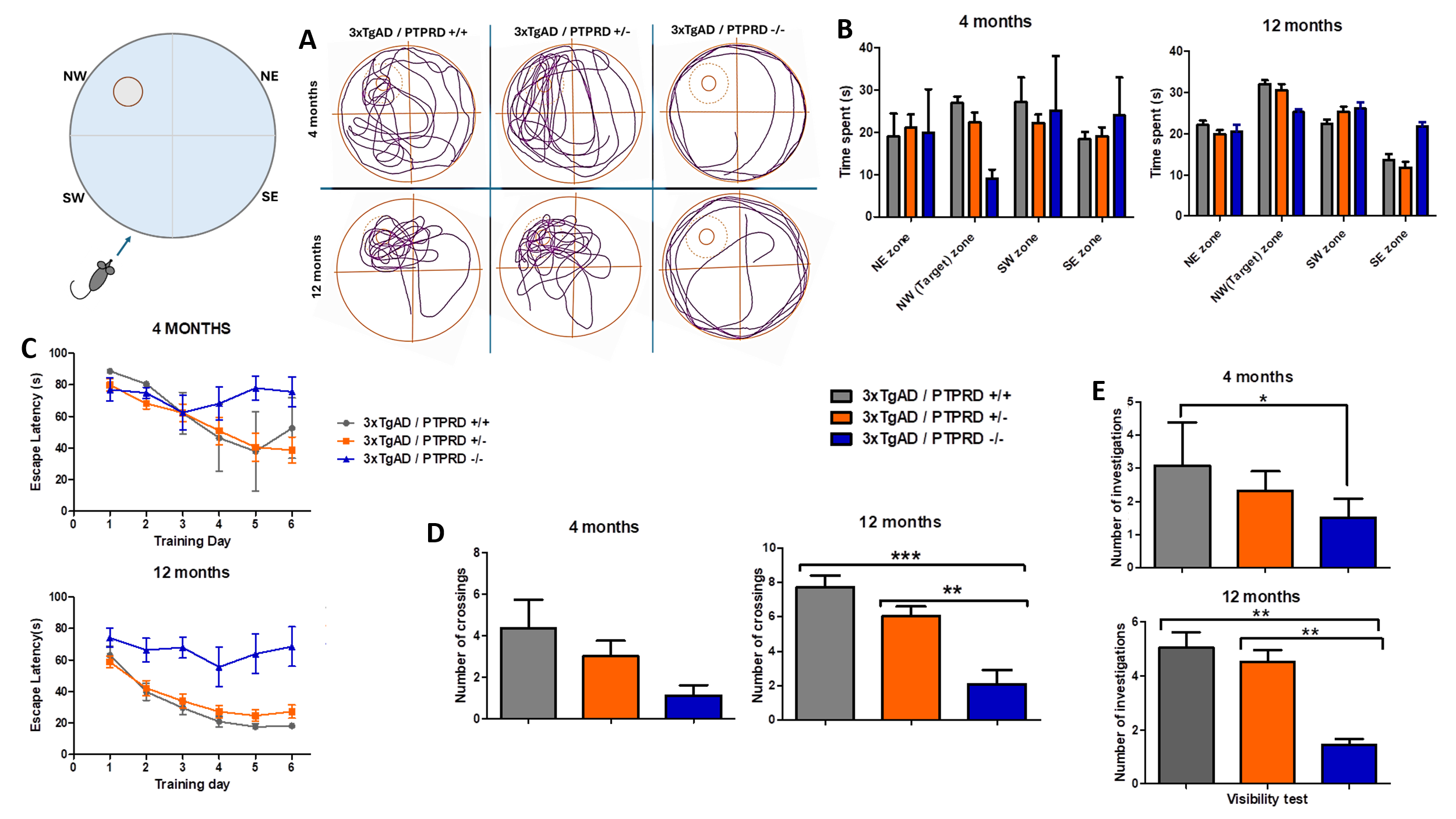

Supplement: Supplementary file 1 [file Data_sheet_1.docx]
